# Variation and Environment in Disease

**Published:** 1911-02-25

**Authors:** 


### Variation and Environment in Disease.

The investigation of the variations in type of certain diseases has not been an unfruitful subject for research, though the inquiries have mostly been made in comparatively restricted areas and under relatively slight differences in environment. If extended over a world-wide field we may suppose that a study of the variations of disease resulting from larger variations of environment would increase our knowledge of the unmodified disease. The term "unmodified disease" simply means for us that type familiar as the result of the study of the disease in question among the races of Western Europe.

It is not difficult to imagine that such important natural factors as high altitude or a great increase in the amount of sunshine would produce considerable variations in the type of any particular disease. The changes in the blood and in the metabolic processes produced by high altitudes have been investigated by many workers, and their results show very definite differences from those regarded as normal at the sea-level. Seeing that in pneumonia there is already considerable embarrassment of the circulation due to the changes in the lung tissue and to the toxæmia, may it not be reasonably supposed that, at high altitudes, the course of this disease may be unfavourably affected by one or more of the factors associated with a low barometric reading—for example, the increased viscosity of the blood due to the polycythæmia?

Another prominent factor in environment is the relative amount of sunshine and its intensity. Here it is difficult to distinguish clearly between the physiological and the pathological. The formation of pigments in the skin on exposure to sunshine may be protective and physiological; on the other hand, a pathological dermatitis is easily caused by actinic light. There are many skin diseases which are admitted to be mainly due to actinic radiation, and, moreover, all of them appear to affect blondes more readily than brunettes or coloured people. It has been suggested that psoriasis is an expression of the resentment of the skin against partial or total exclusion of light, and hence should be relatively commoner in miners and those who are much within doors. Statistical examination is urgently needed of the proposition that the stock of the fair-haired, blue-eyed man from North-West Europe speedily dies out when he attempts to colonise tropical or sub-tropical lands. If on investigation we find that a third or fourth generation of blondes is rare in such a country, then an inquiry into the diseases which destroy the blonde stock would become one of prime political importance.

We may confidently expect that much of our future advance in knowledge of the ætiology of such diseases as duodenal ulcer, osteo-arthritis, cancer, rickets, and rheumatic fever, to quote only a few, will be derived from observations made in those growing centres of civilisation on the African and Australian continents.

When once it can be definitely proved that a disease of obscure ætiology is more severe in type and more common (or the reverse) in certain areas, such a clue may form a starting-point from which the pathology of the disease may gradually be unravelled.

The study of the question of racial liability has hitherto only been investigated to a limited extent, and there is still a want of exact knowledge, due in large measure to the inconvenience of post-mortem examinations in hot countries; but the significance of racial liability to disease seems so great that its investigation justifies some special and systematic undertaking. The whole question of the influences of the many factors included in the term "environment" offers a wide and fertile field for research.

